# Discovering the nucleus in a world of biomaterials

**DOI:** 10.1016/j.bbiosy.2024.100096

**Published:** 2024-06-08

**Authors:** Steven Vermeulen, Elizabeth Rosado Balmayor

**Affiliations:** a MERLN Institute for Technology-Inspired Regenerative Medicine, Maastricht University, Maastricht, the Netherlands; b Experimental Orthopaedics and Trauma Surgery, Department of Orthopaedic, Trauma, and Reconstructive Surgery, RWTH Aachen University Hospital, Aachen, Germany; c Musculoskeletal Gene Therapy Laboratory, Rehabilitation Medicine Research Center, Mayo Clinic, Rochester, MN 55905, USA

**Keywords:** Biomaterial-driven nuclear alterations, Mechanobiology, Surface topography, Advanced technologies

## Abstract

The nucleus serves as the central hub for cellular activity, driving cell identity and behavior. Despite its crucial role, understanding how biomaterials influence the nucleus remains an underexplored area of research. In our opinion, this is an overlooked opportunity, particularly in regenerative medicine — a field where cellular control is not just beneficial, but essential. As such, we emphasize the need to recognize nuclear characteristics as a key metric for evaluating material functionality. In this leading opinion article, we discuss how state-of-the-art technologies can help reveal biomaterial-driven nuclear alterations, offering crucial insights that will advance the field of regenerative medicine.

## Highlights

• The nucleus is a dynamic regulatory hub where epigenetic mechanisms and structural architecture converge to regulate cellular function.
• Culturing cells on biomaterials results in profound epigenetic changes. The full implications of these biomaterial-induced nuclear changes remain unknown.
• There is a need to investigate how biomaterials modulate subnuclear structures and epigenetic profiles to aid our comprehension of the cellular responses elicited by biomaterials.
• Advanced technologies that allow to explore the nucleus in detail, including its DNA, RNA, and protein profiles, are crucial to understand the epigenomic impact of biomaterials.

## The dynamic nucleus

During development, precise regulation of cellular processes is imperative for multiple biological functions, including cell differentiation, cellular activity, and responsiveness to environmental cues. These processes are controlled by modulating nuclear compartments, particularly by epigenetically modifying the genome. Epigenetic regulation encompasses altering DNA methylation patterns and the histone code, with specific modifications including acetylation, methylation, and propionylation; all influencing gene activity. These changes are, in part, irreversible, allowing cells to maintain their identity, while the reversible characteristic is relevant to responding to the environment's needs [1].

In addition to the aforementioned epigenetic mechanisms, recent years have underscored the profound impact of chromatin's structural architecture on transcriptional regulation. Transcriptionally active regions tend to be spatially clustered in dynamic hubs of gene expression. In contrast, transcriptionally inactive regions are often sequestered in the nuclear and nucleolar periphery, organized into lamina or nucleolar-associated domains (LADs or NADs) [2]. Through such spatial organization, the cell exerts another layer of epigenetic control, influencing the accessibility of transcriptional machinery to different genomic regions and thereby modulating gene activity.

In this nuclear landscape, it is not only epigenetic modifications that determine cell behavior. Various nuclear compartments are dynamically regulated to fulfill the phenotypical requirements of the cell or to respond to environmental stimuli. These nuclear compartments include, for example, nucleoli, Cajal bodies, and paraspeckles [3]. These compartments play essential roles in cell function. For example, the nucleolus, often considered the cell's ribosome factory, is essential for synthesizing ribosomal RNA (rRNA) and assembling ribosomal proteins. This compartment is a key regulator of metabolic rate and proliferative capacity, as its activity is closely linked to cell cycle progression. The nucleolus is also important in orchestrating the cellular response to environmental stress. Similarly, other subnuclear compartments act as important gene expression regulators. For example, Cajal bodies are associated with RNA metabolism and manage the maturation and assembly of ribonucleoprotein particles (RNPs) involved in transcription regulation. Other compartments, such as nuclear speckles, are associated with RNP's and are usually found in transcriptionally active genomic regions to facilitate pre-mRNA processing.

In summary, the nucleus is a dynamic regulatory hub where epigenetic mechanisms and structural architecture converge to regulate cellular function. As such, the ability to control this dynamic nature is of great importance to influence cell behavior in vitro. Here, we emphasize that this can be achieved by tailoring cell-biomaterial interactions in order to accommodate a desired application.

## Biomaterials induce profound nuclear alterations

Recent scientific developments have underscored the significant role of biomaterials as important tools in regenerative medicine. These materials actively influence cell behavior, including the modulation of growth factor secretion, stem cell differentiation, and the phenotypic preservation of various cell types [4]. That biomaterials improve the cell culture environment is no surprise considering that traditional cell culture techniques employ flat polystyrene surfaces, which fail to replicate the complex conditions present within the human body. In contrast, biomaterials offer a more physiological milieu by engaging cells with biochemical and/or biomechanical stimuli. Biochemical stimuli can arise from the integration of matrix components that activate integrin or growth factor signaling pathways, particularly when growth factors are incorporated into the matrix [4].

Next to biochemical stimulation, biomaterials have a profound capacity to transduce biomechanical cues. This is made possible through unique design architectures, such as scaffolds, pits, pillars, and curvature over varied scales, as well as chemical modifications, influencing important mechanosensitive pathways [4]. Examples include the MAPK pathway through altered integrin signaling, and the Rho/ROCK/SRF and YAP/TAZ signaling pathways through altered cytoskeleton organization. These signaling events significantly impact essential cell behavior such as cell differentiation [4].

A crucial yet underappreciated aspect of the resulting cellular morphological transformations is however their impact on nuclear shape. There is a strong correlation between cell and nuclear morphology; as a cell contracts, so does its nucleus [5]. Studies have indeed hinted that profound changes occur on the epigenome level when cells are cultured on biomaterials. For example, nano-topographies in the form of nanotubes influence cell morphology and subsequently affect histone regulatory proteins, such as histone methylases and histone deacetylases [6]. Similarly, micro-topographies, which induce strong cell and nuclear confinement, have been shown to cause alterations in histone methylation and acetylation, as well as changes in subnuclear compartments such as the nucleoli [7]. The precise mechanisms by which biomaterials induce epigenetic changes and alter nuclear morphology remain an active area of research.

**Fig. 1:**
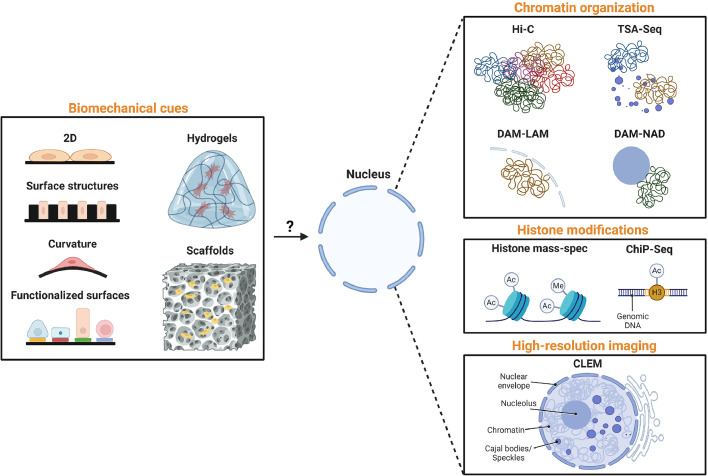
An overview of techniques to elucidate fundamental changes in nuclear biology elicited by biomaterials.

Of interest here is that the broader implications of biomaterial-induced nuclear changes are still unknown, and many fundamental questions remain unanswered. Which genes are affected by these histone modifications? Does culturing cells from 2D to 3D also profoundly impact the 3D conformation of chromatin, thus leading to altered gene expression? Answering these questions can allow us to predict how cells will behave on biomaterials. To achieve this, we need to leverage state-of-the-art technologies, typically reserved for monolayer cultures, to unravel new fundamental insights into how biomaterials transduce their biomechanical cues (Fig. 1; Table 1).

**Table 1:** Summary of proposed technologies for profiling epigenetic and structural changes in the nucleus.

Technology	Metric
Histone proteomics	Global genomic histone profiling
Chip-Seq	Histone profile of individual genes
Hi-C	Structural organization of the genome
DamID	Identification of genomic regions near subnuclear locations
TSA	Identification of genomic regions near subnuclear locations
CLEM	Visualization of subnuclear architectures

## Advanced technologies to understand the epigenomic impact of biomaterials

The influence of biomaterials on the epigenetic landscape, such as influencing histone acetylation and methylation, impacts cell behavior. Of interest is that compared to a flat surface, biomaterials can either increase or decrease acetylation events. Notably, culturing fibroblasts on microgroove surfaces is associated with increased acetylation that enhances cellular reprogramming efficiency towards induced pluripotent stem [8]. This is in contrast with other biomaterials such as micro-topographies that decrease histone acetylation, a phenomenon that is linked with a lower proliferation capacity [7]. A crucial yet unaddressed aspect however is identifying which genes are transcriptionally regulated by these histone modifications. Understanding this relationship is essential to gain deeper insights into how biomaterials influence cellular phenotype.

Exploring each histone modification independently presents a significant challenge, given the existence of over 100 known modifications [1]. A technical solution to this problem can be found by applying histone mass spectrometry [7]. This technique allows quantifying multiple histone modifications in one sample. While this technique cannot measure all histone modifications, it does allow profiling a representative sample of histone modifications induced by biomaterials and might serve as a foundation for subsequent methodologies like chromatin immunoprecipitation (ChIP) assays [9]. By incorporating antibodies specific to biomaterial-altered histones, ChIP assays have the potential to isolate and sequence genes linked to biomaterial-induced epigenetic alterations. As such, we believe that histone mass spectrometry and ChIP assays are powerful tools to understand the epigenetic changes imposed by biomaterials.

Next to histone modifications, the possible impact of biomaterials on chromatin structure and their effect on transcriptional regulation warrants investigation, considering the significant effect biomaterials can exert on nuclear morphology. Here, High-throughput Chromosome Conformation Capture (Hi-C) could prove valuable to achieve this by sequencing cross-linked, digested chromatin fragments, enabling investigating the proximity of genomic locations [9]. Next to Hi-C, other methodologies allow determining chromatin structure, specifically regarding the proximity towards subnuclear structures. A possible methodology is the DamID technology, which allows mapping where a specific protein tags genomic regions, which can then be sequenced. This can be applied for determining genomic interaction sites with lamins (LADs) or nucleoli (NADs). Next to this, also the tyramide signal amplification sequencing (TSA-seq) technology can be ideal for determining the proximity of genomic regions to subnuclear structures such as nuclear speckles [9]. As such, the Hi-C, DamID, or TSA technologies are ideal methods for assessing how biomaterial-induced changes in nuclear architecture affects the structural organization of the genome.

We previously discussed that next to epigenetic regulations, subnuclear structures such as the nucleoli play important roles in cellular function, which can be affected by biomaterials [7]. To further investigate this phenomenon, cutting-edge techniques that visualize and characterize these structures are essential. Such an example is correlative light and electron microscopy (CLEM), which combines fluorescent microscopy with high-resolution ultrastructure imaging provided by electron microscopy [10]. This technology has only rarely been used in the context of biomaterials and it would be particularly interesting to combine this technology with the previously mentioned genomic assays.

## Final remark

In summary, investigating how biomaterials modulate subnuclear structures and epigenetic profiles is crucial for a deeper comprehension of the cellular responses elicited by biomaterials. We urge the scientific community to integrate cutting-edge technological approaches when applying biomaterials to enhance our knowledge of the underlying principles in biomaterial-driven cellular behavior.

## CRediT authorship contribution statement

**Steven Vermeulen:** Writing – original draft, Visualization, Resources, Methodology, Investigation, Funding acquisition, Conceptualization. **Elizabeth Rosado Balmayor:** Writing – review & editing, Visualization, Supervision, Resources, Funding acquisition, Conceptualization.

## Declaration of competing interest

The authors declare that they have no known competing financial interests or personal relationships that could have appeared to influence the work reported in this paper.

## Data Availability

No data was used for the research described in the article.
